# MicroRNA expression signatures associated with metastatic progression in papillary thyroid carcinoma

**DOI:** 10.20945/2359-4292-2026-0058

**Published:** 2026-06-08

**Authors:** Vinicius Ferreira Capelli, Ana Kober Leite, Kelly Cristina Saito, Thérèse Rachell Theodoro, Fátima Solange Pasini, Venâncio Avancini Ferreira Alves, Luiz Paulo Kowalski, Maria Aparecida Silva Pinhal, Edna Teruko Kimura, Leandro Luongo Matos

**Affiliations:** 1 Faculdade de Medicina Santa Marcelina, São Paulo, SP, Brasil; 2 Departamento de Cirurgia de Cabeça e Pescoço, Instituto do Câncer do Estado de São Paulo, Hospital das Clínicas da Faculdade de Medicina da Universidade de São Paulo, São Paulo, SP, Brasil; 3 Faculdade Israelita de Ciências da Saúde Albert Einstein, Hospital Albert Einstein, São Paulo, SP, Brasil; 4 Departamento de Biologia Celular e do Desenvolvimento, Instituto de Ciências Biomédicas, Universidade de São Paulo, São Paulo, SP, Brasil; 5 Departamento de Saúde Pública, Faculdade de Medicina do ABC, São Paulo, SP, Brasil; 6 Centro de Investigação Translacional em Oncologia, Instituto do Câncer do Estado de São Paulo Paulo (ICESP), Hospital das Clínicas (HCFMUSP), Faculdade de Medicina da Universidade de São Paulo, São Paulo, SP, Brasil; 7 Departamento de Patologia, Instituto do Câncer do Estado de São Paulo, Laboratório de Investigação Médica 14 (LIM14), Faculdade de Medicina da Universidade de São Paulo, São Paulo, SP, Brasil; 8 Departamento de Bioquímica, Faculdade de Medicina do ABC, São Paulo, SP, Brasil

**Keywords:** Papillary thyroid carcinoma, microRNAs, metastasis, death

## Abstract

**Objective:**

The objective of this study is to compare the miRNA expression profiles of primary tumors and matched metastatic lesions from patients who died due to progression of metastatic PTC.

**Subjects and methods:**

We conducted an exploratory study of patients with PTC who died from disease progression and had tissue samples available from both primary tumors and distant metastases. Total RNA was extracted and analyzed to assess the expression of 64 preselected miRNAs. Expression data were normalized using endogenous controls (let-7g-5p and miR-181a-5p), and differential expression was calculated using the 2^-∆Ct^ method. Those miRNAs detected in < 70% of samples or with cycle threshold (Ct) > 36 were excluded. Univariate analyses were performed using paired tests, and multivariate results were adjusted for multiple comparisons using the Benjamini-Hochberg false discovery rate (FDR) method.

**Results:**

Out of 3,555 patients treated for PTC between 1986 and 2015, eight patients were included. Univariate analysis identified five miRNAs differentially expressed in metastatic lesions: let-7e-5p, miR-10b-5p, miR-30e-3p, miR-423-5p, and miR-483-3p. After multivariate adjustment, miR-10b-5p and miR-30e-3p remained independently overexpressed in metastatic tissues.

**Conclusion:**

This study is one of the first to demonstrate distinct miRNA expression profiles in metastatic versus primary tumors in fatal PTC cases. The identified miRNAs are known to regulate processes such as cell migration, invasion, and apoptosis in other cancers, suggesting their potential contribution to PTC metastasis.

## INTRODUCTION

Papillary thyroid carcinoma (PTC) is the most prevalent form of thyroid cancer and is typically associated with excellent prognosis and indolent clinical behavior. However, a small subset of cases develops distant metastases, which account for most disease-related mortality (^1^). Despite advances in the understanding of common genetic alterations (^2^), the molecular mechanisms that differentiate indolent from aggressive PTC, particularly in cases that progress to distant metastasis, remain insufficiently understood (^3^).

Although genetic mutations have been extensively studied, the role of microRNAs (miRNAs) in the progression of PTC is an area of emerging research. These small non-coding RNAs play critical roles in gene regulation and have been implicated in various stages of tumor development, invasion, and metastasis across several cancer types (^4^). While dysregulated miRNA expression in thyroid tumors compared to normal tissue has been reported (^3^), few studies have specifically investigated their involvement in metastatic dissemination and poor clinical outcomes in solid malignancies, and none have addressed this question directly in PTC.

The objective of this study was to compare the miRNA expression profiles of primary tumors and matched metastatic lesions from patients who died due to progression of metastatic PTC. By identifying differentially expressed miRNAs associated with distant disease, we aim to provide insights into the biological processes underlying metastatic progression and contributing to mortality in this typically indolent malignancy. This study was conducted using paired formalin-fixed paraffin-embedded (FFPE) samples from a cohort of patients with PTC evaluated and treated at the Head and Neck Surgery Service of the Instituto do Câncer do Estado de São Paulo (ICESP) and Hospital das Clínicas, Faculdade de Medicina da USP (HC-FMUSP).

## SUBJECTS AND METHODS

This study was approved by the Institutional Ethics Committee (CAAE: 44997215.1.0000.0065). Patients included had available samples from both the primary tumor and any metastatic tissues. The tissue samples used for molecular analysis consisted of a small portion of tumor remnants, selected by microdissection and embedded in FFPE blocks, which were originally collected for histopathological examination; this procedure ensured that only tumor tissue fragments were analyzed.

### Sequencing for *BRAF* and *TERT* mutations and *NTRK* fusion

Genomic DNA was extracted from the FFPE samples using a standardized protocol. Nested PCR was used to amplify the *BRAF* and *TERT* promoter gene regions. Specific primers were designed using the Primer-BLAST tool. The PCR products were visualized by gel electrophoresis, purified, and then subjected to capillary sequencing using the Sanger method. Sequence analysis and mutation identification were performed using Sequence Scanner software (Applied Biosystems, Thermo Fisher Scientific, Waltham, MA, USA) and BLAT database alignment. Additionally, all samples underwent next-generation sequencing using the Ion Torrent platform (Thermo Fisher Scientific). Data were processed on the Ion Torrent Server and annotated via the Oncomine Knowledge Base (Thermo Fisher Scientific). The sequencing panel was designed to detect gene fusions involving *NTRK1, NTRK2*, and *NTRK3*, including known fusion partners for each gene. These techniques have been employed in previously published studies of our group (^2^,^3^).

### MicroRNA detection technique

A total of 64 miRNAs were preselected for analysis based on a comprehensive literature review, as previously described (^3^). These miRNAs were selected because they have been previously associated with tumor aggressiveness, epithelial-mesenchymal transition (EMT), metastatic spread, or prognosis in different cancer types, including thyroid cancer pathogenesis and metastatic behavior. Total RNA was extracted from ten 5-µm sections of FFPE tissue using the MagMAX FFPE RNA Ultra Kit (Thermo Fisher Scientific). Expression levels of selected miRNAs were quantified using reverse transcription followed by quantitative PCR (RT-qPCR), employing the TaqMan Low Density Array (TLDA) platform (Applied Biosystems). The sequences of all 64 selected miRNAs are demonstrated in **Supplementary Table 1**.

Each sample was analyzed in triplicate to ensure consistency. Cycle threshold (Ct) values greater than 36 were excluded as unreliable, and only miRNAs expressed in at least 70% of the samples were included for downstream analysis, following the validated pipeline from our previous study (^3^). This filtering step led to the exclusion of 18 miRNAs from the original panel (miR-129-5p, miR-130b-3p, miR-137-3p, miR-138-2-3p, miR-146b-3p, miR-155-3p, miR-17-3p, miR-187-3p, miR-302c-3p, miR-30e-5p, miR-34b-3p, miR-34c-5p, miR-455-3p, miR-4788, miR-506-3p, miR-654-3p, miR-9-5p, and miR-98-5p).

For normalization, the quantile method was applied using Expander software, selecting the most stable endogenous miRNAs as references. Specifically, the average expression of let-7g-5p and miR-181a-5p was used as an internal control, selected based on their stability after quantile normalization. The relative expression of each miRNA was calculated using the 2^-∆Ct^ method, enabling the identification of differentially expressed miRNAs between the two patient groups.

### Statistical analysis

Categorical data were described as frequencies. Continuous variables were reported as means (standard deviations [SD] or standard errors [SE]). The ranking of paired tissues in the miRNA profile analysis was evaluated using the Wilcoxon test. Variables with p-values < 0.10 in univariate analysis were further assessed using multivariate linear regression to identify independently associated miRNAs, given the very small sample size (8 pairs), which does not support stable estimation in mixed-effects frameworks. To correct for multiple comparisons, p-values were adjusted using the Benjamini-Hochberg false discovery rate (FDR) method. An FDR-adjusted p-value (q-value) < 0.05 was considered statistically significant. All analyses were conducted using SPSS v29.0 (IBM Corp., Armonk, NY, USA). A graphical representation of differential miRNA expression was produced with assistance from ChatGPT (OpenAI) using Python, with code generated and refined by the authors. In this visualization, bubble size reflected statistical significance using the -log10 (FDR) transformation, and bubble color encoded the β coefficients from the multivariate model.

## RESULTS

Out of a total of 3,555 patients diagnosed with PTC and treated from 1986 to 2015, 108 (3%) were identified as having distant metastasis, either at the initial PTC diagnosis or during follow-up. From this cohort, we selected eight patients who died due to disease progression and had available tissue from both the primary tumor and metastatic sites, with a mean time from diagnosis to death of 44 months (SD 12.6 months). The majority were women (75%) with a mean age of 55.2 years (SD 9.6 years). All patients underwent R0 surgery, and 87.5% received radioiodine therapy. Half of the patients developed radioiodine-refractory disease, and 75% had evidence of vascular invasion. All patients exhibited distant metastases-most commonly to the lung (7; 87.5%), bone (7; 87.5%), or multiple sites (7; 87.5%). Three patients (37.5%) had *BRAF* mutation detected at the primary tumor and also at the metastatic tissue. Four patients (50%) exhibited *TERT* mutations, including the same three patients with *BRAF* mutations, in both primary and metastatic specimens. One patient had an exclusive *TERT* mutation detected only on metastatic tissue, while no samples displayed *NTRK* fusions. The complete descriptive data are available in **Table 1**.

**Table 1:** Descriptive data of patients with metastatic papillary thyroid carcinoma who died due to disease progression and were included in the study

Categorical variables	Frequency (n)		Valid %		
Sex					
Male	2		25		
Female	6		75		
R0 Surgery^*^	8		100		
Reoperation	2		25		
Radioiodine Therapy (RAI)	7		87.5		
Stimulated Thyroglobulin (> 4 ng/mL)	8		100		
Radioiodine Refractoriness	4		50		
Lymph Node Recurrence	2		25		
Site of Distant Metastasis					
Lung	7		87.5		
Liver	2		25		
Bone	7		87.5		
Multiple Sites	7		87.5		
Cause of Death					
Airway Obstruction	3		37.5		
Metastatic Tumor Progression	5		72.5		
Aggressive Histological Variant	1		12.5		
TNM 8th Edition - pT					
pT1	3		37.5		
pT2	2		25		
pT3	1		12.5		
pT4a	2		25		
TNM 8th Edition - pN					
pN0	4		50		
pN1a	2		25		
pN1b	2		25		
Vascular Invasion	6		75		
Extrathyroidal Extension	4		50		
Microscopic	1		12.5		
Macroscopic	3		37.5		
Multifocality	5		62.5		
Dedifferentiation	5		62.5		
Mutation Profile					
*BRAF* (Primary Tumor)	3		37.5		
*BRAF* (Metastatic Tissue)	3		37.5		
*TERT* (Primary Tumor)	4		50		
*TERT* (Metastatic Tissue)	5		62.5		
*NTRK* Fusion (Primary Tumor)	0		0		
*NTRK* Fusion (Metastatic Tissue)	0		0		
Continuous variables	Minimum	Maximum		Mean	SD
Age (years)	42	72		55.2	9.6
Cumulative Radioiodine Dose (mCi)	200	720		349	169.4
Tumor Size (cm)	0.4	13.5		3.6	4.5
Time from Diagnosis to Death (months)	30	67		44	12.6
Time from Diagnosis to Metastasis (months)	0	13		1.9	4.6
Time from Diagnosis to Disease Progression (months)	13	61		27	16.4
Time from Metastasis to Death (months)	22	67		42.1	14.7
Time from Disease Progression to Death (months)	3	41		17	11.7

* R0 surgery: complete resection with negative margins.

SD: standard deviation.

Remarkably, miRNAs let-7e-5p, miR-10b-5p, miR-30e-3p, miR-423-5p, and miR-483-3p were overexpressed in metastatic tissues compared to primary tumor tissues, as shown in **Table 2**.

**Table 2:** Comparison of microRNA expression between primary tumor and metastatic tissue

miR-10b-5p	Primary tumor		Metastatic tissue		*P*-value (Wilcoxon test)
	Mean	SE	Mean	SE	
let-7b-5p	0.02886	0.011184	0.33854	0.174389	0.093
let-7c-5p	0.02886	0.276381	2.07338	0.88759	0.465
let-7d-5p	0.02886	0.039087	0.76483	0.260128	0.249
*let-7e-5p*	*0.02886*	*0.215269*	*3.77295*	*0.735996*	*0.036*
let-7f-5p	0.02886	0.562676	4.28175	1.142207	0.753
let-7i-5p	0.02886	4.832131	15.96943	7.14814	0.600
miR-1-3p	0.02886	1.212567	19.44099	9.018971	0.063
*miR-101-3p*	*4.23332*	*0.810301*	*3.19856*	*0.649048*	*0.398*
*miR-10b-5p*	*0.37133*	*0.069863*	*2.23117*	*0.807449*	*0.018*
miR-125a-5p	6.30632	1.241309	8.45051	2.054138	0.208
miR-138-5p	3.44566	1.271794	4.09477	2.284513	1.000
miR-141-3p	14.46202	3.71996	20.58323	6.642364	0.893
miR-16-5p	5.6556	0.747569	8.40206	3.212635	0.398
miR-17-5p	2.2212	0.73003	1.96044	0.701114	1.000
miR-181b-5p	0.25988	0.062959	0.44196	0.088307	0.345
miR-18a-5p	0.05851	0.03011	0.18816	0.098839	1.000
miR-191-5p	1.02326	0.247608	1.34079	0.2897	0.735
miR-199a-3p	1.02775	0.346961	0.40159	0.183531	0.310
miR-19a-3p	0.76139	0.382344	0.46611	0.126985	0.753
miR-19b-3p	1.45986	0.687182	1.70509	0.574711	0.500
miR-200a-3p	2.52547	0.654323	3.09188	0.916931	0.889
miR-200b-3p	30.90601	10.271064	23.8411	9.510103	0.327
miR-200c-3p	13.44791	4.665669	27.43071	8.587642	0.123
miR-203a-3p	0.09001	0.048652	0.03674	0.021409	0.109
miR-205-5p	1.64797	1.369495	3.41519	2.121461	1.000
miR-20a-5p	0.63947	0.212446	0.66561	0.129087	0.735
miR-21-5p	45.36348	12.57295	42.09528	11.896246	0.866
miR-214-3p	0.32772	0.142964	0.22648	0.116418	0.310
miR-221-3p	18.86526	6.648546	20.11025	6.30474	0.575
miR-222-3p	1.04256	0.350446	1.43419	0.354886	0.327
miR-29a-3p	6.23873	0.48776	6.73183	1.380566	0.674
miR-30a-5p	4.96206	0.592394	5.99355	0.875202	0.345
miR-30b-5p	17.94068	3.919451	13.94327	4.067131	0.612
miR-30c-5p	33.66616	6.438056	31.07315	17.379167	0.463
miR-30d-5p	2.20432	0.455019	1.52911	0.534786	0.398
*miR-30e-3p*	*0.58264*	*0.092873*	*1.43238*	*0.276001*	*0.028*
miR-31-5p	0.06265	0.042879	0.41265	0.205599	0.273
miR-34a-5p	3.09104	0.778726	6.54144	2.432161	0.063
*miR-423-5p*	*0.3717*	*0.080369*	*1.48887*	*0.337737*	*0.046*
miR-429	0.12836	0.040788	0.22871	0.08896	0.225
*miR-483-3p*	*0.13068*	*0.04256*	*0.59526*	*0.152839*	*0.043*
miR-92a-3p	11.7837	4.260977	12.08097	4.242697	0.866
miR-146b-5p	1.40705	0.553416	1.91032	1.169727	0.345

SE: standard error.

**Figure 1:**
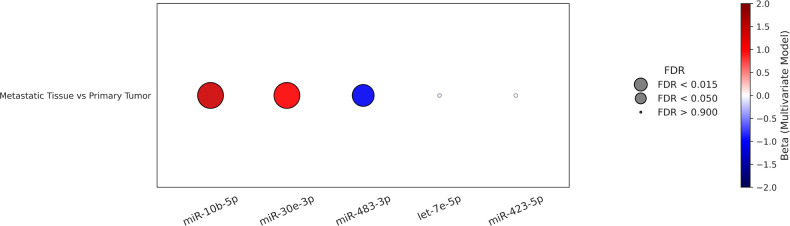
Multivariate association of selected microRNAs in metastatic tissue compared with primary tumors of patients with papillary thyroid carcinoma. Bubble color represents the direction and magnitude of the association based on regression coefficients (β) from the multivariate linear regression model, with positive coefficients shown in red and negative coefficients in blue. Bubble size is proportional to statistical significance and reflects -log10(FDR). While miR-10b-5p and miR-30e-3p showed positive independent coefficients, indicating an association with metastatic tissue, miR-483-3p exhibited a negative independent coefficient after multivariate adjustment. Notably, let-7e-5p and miR-423-5p did not retain statistical significance after FDR correction and are displayed with reduced opacity.

The five differentially expressed miRNAs identified in univariate analysis were subsequently subjected to multivariate linear regression analysis. The resulting p-values were adjusted for multiple comparisons using the FDR method. After adjustment, three miRNAs remained independently and significantly associated with metastatic tissue in the multivariate model. Specifically, miR-10b-5p (β: +1.376; 95% confidence interval [CI]: 0.260 to 0.953; p = 0.006) and miR-30e-3p (β: +1.017; 95% CI: 0.593 to 1.775; p = 0.004) showed positive independent coefficients, indicating an association with metastatic tissue. In contrast, miR-483-3p exhibited a negative independent coefficient (β: -1.078; 95% CI: -2.830 to -0.226; p = 0.030), despite its higher expression in metastatic tissue in univariate analysis, suggesting that the apparent upregulation of miR-483-3p reflects shared variance with other metastasis-associated miRNAs, rather than an independent metastatic signal. Notably, let-7e-5p (β: -0.173; p = 0.887) and miR-423-5p (β: +0.054; p = 0.916) did not retain statistical significance after adjustment. These results are summarized in a bubble plot demonstrated in **Figure 1**.

## DISCUSSION

The molecular mechanisms that differentiate indolent from aggressive PTC, particularly at the metastatic stage, are still poorly understood. Although miRNAs may contribute to the metastatic cascade, comparative analyses between primary tumors and matched metastatic lesions in lethal cases are notably lacking. The present study is part of a broader investigation previously published by our group (^3^), which aimed to identify miRNAs associated with disease-specific mortality in patients with metastatic PTC. In that study, we analyzed samples from 24 patients and demonstrated that the overexpression of miR-101-3p, miR-17-5p, and miR-191-5p was significantly associated with death due to disease progression. From that original cohort, we specifically selected eight patients with available paired samples from both the primary tumor and metastatic sites, which constituted the final study population of the present analysis. The current analysis builds upon those findings by further exploring the functional relevance of selected miRNAs within the context of aggressive PTC biology, reinforcing their potential role in metastatic progression.

In this exploratory study, we found that miR-10b-5p and miR-30e-3p were significantly overexpressed in metastatic tissues compared to their matched primary tumors. Moreover, the divergence between univariate and multivariate results for miR-483-3p likely reflects shared variance with other metastasis-associated miRNAs, particularly given the small sample size, and should therefore be interpreted as a context-dependent rather than an independent effect. Each of these miRNAs has been previously linked to metastatic behavior in other tumor types.

For instance, miR-10b-5p has been linked to the promotion of migration and invasion, with consistent associations with metastasis in breast, gastric, and hepatocellular carcinomas, reinforcing its potential relevance in PTC dissemination (^5^-^7^). Additionally, miR-10b-5p has been consistently implicated in the enhancement of tumor invasiveness and metastatic potential across several malignancies, providing mechanistic clues that may be relevant to metastatic dissemination in PTC. In gastric cancers, miR-10b promotes cellular migration and invasion largely through suppression of tumor-suppressive transcription factors such as HOXD10, thereby releasing downstream prometastatic effectors, including RhoC and matrix-remodeling enzymes that facilitate EMT and tissue invasion (^6^). In hepatocellular carcinoma, esophageal cancer, and low-grade gliomas, miR-10b-5p has been shown to modulate cell-cycle progression and apoptosis through targeting *KLF4*, contributing to an aggressive phenotype characterized by increased proliferation and migratory capacity (^7^). Evidence from non-small cell lung cancer further supports its role in metastasis: circulating exosomal miR-10b-5p is independently associated with worse survival and is proposed to promote metastatic spread by transferring proinvasive signals to recipient cells and modifying the tumor microenvironment (^5^). Together, these converging findings across tumor types indicate that miR-10b-5p acts as a regulator of pathways involved in cytoskeletal remodeling, EMT induction, apoptosis suppression, and extracellular matrix degradation - biological functions that may plausibly contribute to the metastatic behavior observed in advanced PTC.

Similarly, miR-30e-3p demonstrates a context-dependent dual role in cancer biology, acting either as a tumor suppressor or an oncogenic mediator depending on the molecular background and the signaling pathways engaged. In head and neck squamous cell carcinoma, miR-30e-3p suppresses TGF-β pathway mediators-notably TGFβR1 and BMPR2 - leading to reduced migration, invasion, and enhanced antitumoral immune activation through M1 macrophage polarization (^8^). Additional mechanistic data from gastric cancer indicate that miR-30e-3p can inhibit Snail1, thereby attenuating EMT and metastatic potential (^9^). Taken together, these findings show that miR-30e-3p intersects with multiple pathways relevant to tumor progression-including TP53/MDM2 regulation, PTEN/AKT signaling, TGF-β signaling, and EMT - supporting the possibility that its overexpression in metastatic PTC reflects engagement of similar invasionand survival-related mechanisms.

Furthermore, miR-483-3p, located within the *IGF2* gene locus, has been consistently associated with oncogenesis through antiapoptotic and proliferative mechanisms in several malignancies (^10^-^15^). In colorectal cancer, miR-483-3p has been shown to promote proliferation and migration through the suppression of *DLC-1*, thereby enhancing metastatic potential (^10^) In pancreatic cancer, this miRNA directly inhibits DPC4/Smad4, disrupting TGF-β signaling and facilitating tumor growth and invasion (^11^). Coordinated regulation of *IGF2* and miR-483 family members further contributes to oncogenic signaling and cell-cycle progression in multiple solid tumors (^13^). In pancreatic ductal carcinoma, miR-483-3p has been associated with aggressive clinical behavior through modulation of apoptotic pathways and enhancement of proliferative signaling (^14^). Additionally, miR-483-3p has been reported to be upregulated in treatment-resistant breast cancer models, where its increased expression has been associated with modulation of metastasis-related genes (^15^). Taken together, these mechanisms highlight miR-483-3p as a regulator of apoptosis inhibition, TGF-β pathway disruption, proliferative capacity, and cytoskeletal and migratory remodeling across several cancer types.

In contrast, a previous study suggested a tumor-suppressive role for miR-483-3p, including the regulation of cyclin E1 signaling in breast cancer (^12^). In our cohort, miR-483-3p displayed higher expression in metastatic tissue in univariate paired analysis; however, this signal did not persist as an independent association after multivariate adjustment. Specifically, miR-483-3p exhibited a negative coefficient in the multivariate model, indicating that its apparent upregulation in metastatic lesions was not independent of other miRNAs included in the analysis. This divergence between univariate and multivariate results is most plausibly explained by shared variance among metastasis-associated miRNAs and by coefficient instability inherent to multivariate modeling in very small samples, like ours. Given the expected coexpression and biological interdependence of miRNAs involved in metastatic progression, multivariate adjustment in this context likely partitioned overlapping biological signals across correlated predictors rather than revealing true biological downregulation. Accordingly, the role of miR-483-3p in metastatic PTC should be interpreted as context-dependent rather than as an independent suppressive or promotive marker. These findings underscore the regulatory complexity of miR-483-3p and highlight the need for functional and larger-scale studies to clarify whether its expression reflects adaptive responses within the metastatic microenvironment or indirect modulation of metastatic programs.

The process of metastasis in epithelial tumors, including PTC, involves multiple steps such as EMT, invasion, and extravasation, all of which can be regulated by miRNAs. While some miRNAs have been identified as prometastatic in PTC, others act as metastasis suppressors (^16^).

This study is limited by its small sample size, which reduces statistical power, reflecting the challenge of acquiring high-quality, matched pathological specimens for molecular analysis in advanced PTC. Moreover, because seven of the eight patients received radioactive iodine, three were treated with targeted therapies, and six underwent additional systemic treatments, we cannot exclude the possibility that some of the observed miRNA differences reflect treatment-related molecular alterations rather than metastatic progression alone. Another limitation is that, by prioritizing selected biologically plausible miRNAs, the panel did not include all well-known miRNAs previously associated with PTC. Nonetheless, it represents a pioneering effort to explore miRNA expression directly in metastatic tissues from patients with lethal disease.

Our findings expand this body of knowledge by identifying novel miRNAs - overexpressed miR-10b-5p and miR-30e-3p - specifically in metastatic tissues from patients with lethal disease, underscoring their potential role in the metastatic process. These initial findings suggest that miRNA profiling could enhance our understanding of metastatic progression in PTC and may ultimately support the development of prognostic biomarkers or therapeutic targets. Further large-scale, multicenter studies and functional validations are needed to further elucidate the precise roles of these miRNAs in thyroid cancer metastasis.

## Figures and Tables

**Supplementary Table 1:** Primer sequences for the studied microRNAs

microRNA	Sequence
*let-7b-5p*	UGAGGUAGUAGGUUGUGGUU
*let-7c-5p*	UGAGGUAGUAGGUUGUAUGGUU
*let-7d-5p*	AGAGGUAGUAGGUUGCAUAGUU
*let-7e-5p*	UGAGGUAGGAGGUUGUAUAGUU
*let-7f-5p*	UGAGGUAGUAGAUUGUAUAGUU
*let-7g-5p*	UGAGGUAGUAGUUUGUACAGUU
*let-7i-5p*	UGAGGUAGUAGUUUGUGCUGUU
*miR-1-3p*	UGGAAUGUAAAGAAGUAUGUAU
*miR-101-3p*	UACAGUACUGUGAUAACUGAA
*miR-10b-5p*	UACCCUGUAGAACCGAAUUUGUG
*miR-125a-5p*	UCCUGAGACCCUUUAACCUGUGA
*miR-129-5p*	CUUUUUGCGGUCUGGGGCUUGC
*miR-130b-3p*	CAGUGCAAUGAUGAAAGGGCAU
*miR-137-3p*	UUAUUGCUUAAGAAUACGCGUAG
*miR-138-2-3p*	GCUAUUUCACGACACCAGGGUU
*miR-138-5p*	AGCUGGUGUUGUGAAUCAGGCCG
*miR-141-3p*	UAACACUGUCUGGGUAAAGAUGG
*miR-146b-3p*	UGCCCUGUGGACUCAGUUCUGG
*miR-146b-5p*	UGAGAACUGAAUUCCAUAGGCU
*miR-155-3p*	CUCCUACAUAUUAGCAUUAACA
*miR-16-5p*	UAGCAGCACGUAAAUAUUGGCG
*miR-17-3p*	ACUGCAGUGAAGCACUUGUAG
*miR-17-5p*	CAAAGUGCUUACAGUGCAGGUAG
*miR-181a-5p*	AACAUUCAACGCUGUCGGUGAGU
*miR-181b-5p*	AACAUUCAUUGCUGUCGGUGGGU
*miR-187-3p*	UCGUGUCUUGUGUGCAGCCGG
*miR-18a-5p*	UAAGGUGCAUCUAGUGCAGAUAG
*miR-191-5p*	CAACGGAAAUCCCAAAAGCAGCUG
*miR-199a-3p*	ACAGUAGUCUGCACAUUGGUUA
*miR-19a-3p*	UGUGCAAAUCUAUGCAAAACUGA
*miR-19b-3p*	UGUGCAAAUCCAUGCAAAACUGA
*miR-200a-3p*	UAACACUGUCUGGGUAACGAUGU
*miR-200b-3p*	UAAUACUGCCUGGUAAUGAUGA
*miR-200c-3p*	UAAUACUGCCGGGUAAUGAUGGA
*miR-203a-3p*	GUGAAAUGUUUAGGACCACUAG
*miR-205-5p*	UCCUUCAUUCCACCGGAGUCUG
*miR-20a-5p*	UAAAGUGCUUAUAAGUGCAGGUAG
*miR-21-5p*	UAGCUUAUCAGACUGAUGUUGA
*miR-214-3p*	ACAGCAGGCACAGACAGGCAGU
*miR-221-3p*	AGCUACAUUGUCUGCUGGGUUUC
*miR-222-3p*	AGCUACAUCUGGCUACUGGGU
*miR-29a-3p*	UAGCACCAUCUGAAAUCGGUUA
*miR-302c-3p*	UAAGUGCUUCCAUGUUUCAGUGG
*miR-30a-5p*	UGUAAACAUCCUCGACUGGAAG
*miR-30b-5p*	UGUAAAACAUCCUACACUCAGCU
*miR-30c-5p*	UGUAAACAUCCUACACUCUCAGC
*miR-30d-5p*	UGUAAACAUCCCCGACUGGAAG
*miR-30e-3p*	CUUUCAGUCGGAUGUUUACAGC
*miR-30e-5p*	UGUAAAACAUCCUUGACUGGAAG
*miR-31-5p*	AGGCAAGAUGCUGGCAUAGCU
*miR-34a-5p*	UGGCAGUGUCUUAGCUGGUUGU
*miR-34b-3p*	CAAUCACUAACUCCACUGCCAU
*miR-34c-5p*	AGGCAGUGUAGUAGCUGAUUGC
*miR-423-5p*	UGAGGGGCAGAGAGCGAGACUUU
*miR-429*	UAAUACUGUCUGGUAAAACCGU
*miR-455-3p*	GCAGUCCAUGGGCAUAUACAC
*miR-4788*	UUACGGACCAGCUAAGGGAGGC
*miR-483-3p*	UCACUCCUCUCCUCCCGUCUU
*miR-506-3p*	UAAGGCACCCUUCUGAGUAGA
*miR-654-3p*	UAUGUCUGCUGACCAUCACCUU
*miR-9-5p*	UCUUUGGUUAUCUAGCUGUAUGA
*miR-92a-3p*	UAUUGCACUUGUCCCGGCCUGU
*miR-98-5p*	UGAGGUAGUAAGUUGUAUUGUU

## Data Availability

datasets related to this article will be available upon request to the corresponding author.
